# Reversible Plasticity of Fear Memory-Encoding Amygdala Synaptic Circuits Even after Fear Memory Consolidation

**DOI:** 10.1371/journal.pone.0024260

**Published:** 2011-09-19

**Authors:** Ingie Hong, Jihye Kim, Junuk Lee, Sungmo Park, Beomjong Song, Jeongyeon Kim, Bobae An, Kyungjoon Park, Hyun Woo Lee, Seungbok Lee, Hyun Kim, Sang-Hyun Park, Khee Dong Eom, Sukwon Lee, Sukwoo Choi

**Affiliations:** 1 School of Biological Sciences, College of Natural Sciences, Seoul National University, Seoul, Korea; 2 Division of Brain Korea 21 Biomedical Science, Department of Anatomy, College of Medicine, Korea University, Seoul, Korea; 3 Department of Cell and Developmental Biology, School of Dentistry, Seoul National University, Seoul, Korea; 4 School of Biological Sciences, Nanyang Technological University, Singapore, Singapore; University of New South Wales, Australia

## Abstract

It is generally believed that after memory consolidation, memory-encoding synaptic circuits are persistently modified and become less plastic. This, however, may hinder the remaining capacity of information storage in a given neural circuit. Here we consider the hypothesis that memory-encoding synaptic circuits still retain reversible plasticity even after memory consolidation. To test this, we employed a protocol of auditory fear conditioning which recruited the vast majority of the thalamic input synaptic circuit to the lateral amygdala (T-LA synaptic circuit; a storage site for fear memory) with fear conditioning-induced synaptic plasticity. Subsequently the fear memory-encoding synaptic circuits were challenged with fear extinction and re-conditioning to determine whether these circuits exhibit reversible plasticity. We found that fear memory-encoding T-LA synaptic circuit exhibited dynamic efficacy changes in tight correlation with fear memory strength even after fear memory consolidation. Initial conditioning or re-conditioning brought T-LA synaptic circuit near the ceiling of their modification range (occluding LTP and enhancing depotentiation in brain slices prepared from conditioned or re-conditioned rats), while extinction reversed this change (reinstating LTP and occluding depotentiation in brain slices prepared from extinguished rats). Consistently, fear conditioning-induced synaptic potentiation at T-LA synapses was functionally reversed by extinction and reinstated by subsequent re-conditioning. These results suggest reversible plasticity of fear memory-encoding circuits even after fear memory consolidation. This reversible plasticity of memory-encoding synapses may be involved in updating the contents of original memory even after memory consolidation.

## Introduction

Memory is encoded and consolidated within neural circuits in a protein-synthesis-dependent manner over time [1], [2]. Consolidated memory has been shown to persist across the adult lifetime, which implies that the neural substrate for consolidated memory must be persistent [3]. Indeed, memory consolidation appears to involve the conversion of labile synaptic potentiation into a persistent increase in synaptic efficacy [4]. The belief that such persistent synaptic modifications underlie consolidated memory leads to the assumption that the synapses involved in memory encoding lose plasticity after consolidation and are less modifiable thereafter. Because memories are formed sequentially rather than all at once, this restriction inevitably lessens the capacity of information storage in a given neural circuit. Therefore, the question whether memory-encoding synaptic circuits can be reused has attracted much attention. To date, however, it is yet to be demonstrated that sequential learning can recruit such reversible plasticity of memory-encoding synaptic circuits after memory consolidation, therefore most learning-induced plasticities (i.e. LTP & LTD) are studied separately in different brain regions and learning paradigms.

One reason that the observation of such reversible plasticity has been elusive is that the site of initial neural memory encoding and consolidation can be different, as is the case with memories involving the hippocampus. Several memory tasks that are initially hippocampus-dependent slowly transfer to a hippocampus-independent state, suggesting a transfer of memory locus to cortical sites [5], [6], [7], [8]. Moreover, this consolidation process can continue for days and weeks, rendering it difficult to pinpoint the substrate of consolidated memory. On the contrary, auditory fear memory is consolidated in the lateral amygdala (LA) in a rapid (<24 hrs) and local manner [9], [10], [11]. The potentiation of T-LA synapses, which accompanies fear conditioning, is required for both short-term and long-term fear memory [12], [13], [14], [15]. Moreover, auditory fear memory is maintained in the LA across the adult lifetime of rats [3]. Interestingly, recent reports have suggested that the memory trace in the LA is not completely static. Reactivation of fear memory apparently renders consolidated memory and its trace susceptible to pharmacological disruption [16], [17], while fear extinction, a weakening of conditioned fear memory association, appears to involve a corresponding weakening (depotentiation) of amygdala synapses [18], [19], [20], [21]. Although these studies suggest that fear conditioning-induced potentiation at T-LA synapses can be modified after consolidation, they fall short of addressing whether these synapses can support further plasticity and learning.

We thereby tested the hypothesis that learning can induce reversible plasticity at memory-encoding synapses in the lateral amygdala after consolidation. First we established a method to assess the ceiling and floor of synaptic modification by estimating LTP and depotentiation induction in amygdala slices prepared from behavior-trained rats; 1) no LTP and significant depotentiation in the ceiling, 2) significant LTP and no depotentiation in the floor. We then assessed relationship between input stimulus strength and synaptic output, a direct measure of synaptic efficacy, in amygdala slices prepared from behavior-trained rats. Using these two independent measures, we provide evidence that memory-encoding T-LA synapses retain reversible plasticity even after fear memory consolidation.

## Results

**Figure 1:**
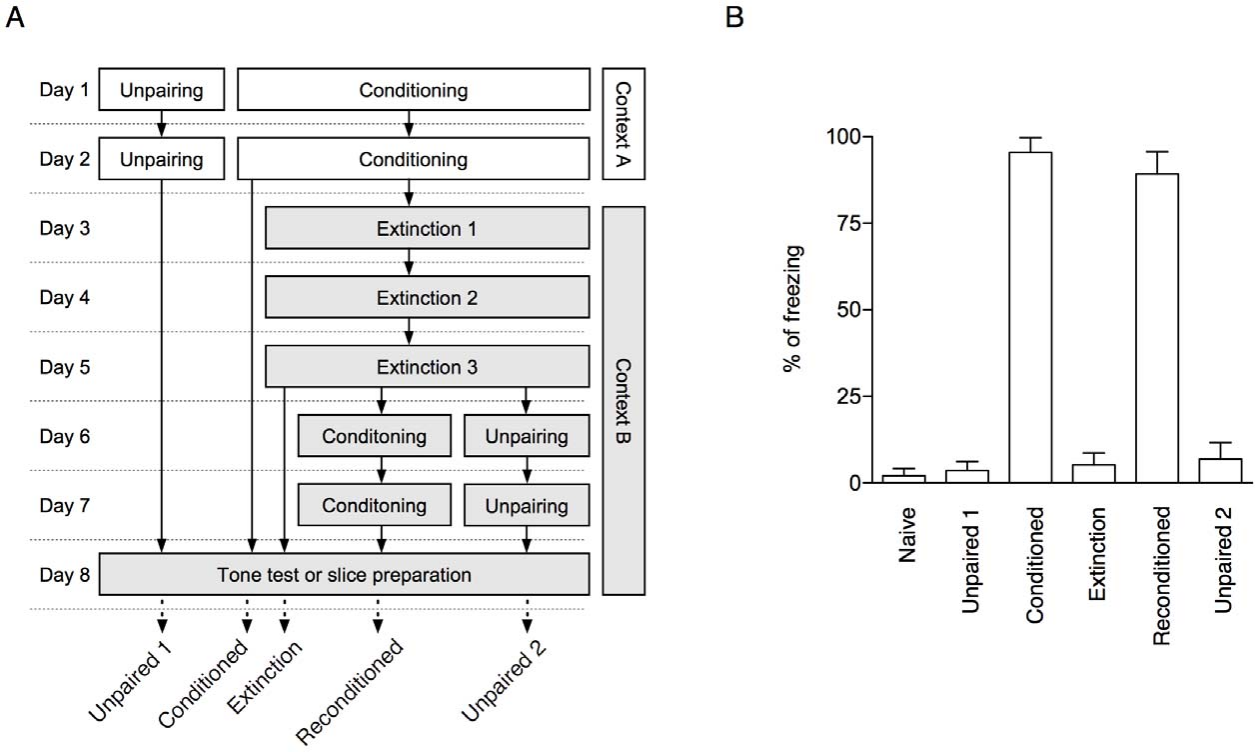
Behavioral procedures. **A.** Schematic diagram for behavioral procedures. Conditioning was carried out for two consecutive days, followed by extinction (three days) and reconditioning (two days) in a distinct context. White and gray tones in the rectangles represent context A and B, respectively. On day eight, brain slices were acquired from rats, while a separate set of animals were tested for fear memory retention. **B.** Pooled behavioral results for Fig. 2 and 3.

We first established a behavioral protocol to test the reversible plasticity of memory-encoding T-LA synaptic circuits (Fig. 1A). A 3-day scheduled extinction training was found to eliminate conditioned freezing induced by six tone-shock pairings, and subsequent reconditioning with six tone-shock pairings was as effective in inducing strong freezing as initial conditioning (naïve, 2.1±2.1%; unpaired 1, 3.6±2.6%; conditioned, 95.5±4.6%; extinction, 5.2±3.5%; reconditioned, 89.2±6.4%; unpaired 2, 6.8±4.8%; F_4,69_ = 118.1, p<0.01; p<0.05 for conditioned or reconditioned vs. the other three groups, Newman-Keuls posttest; Fig. 1B). The unpaired controls for the reconditioned groups (unpaired group 2) were the same as the reconditioned groups except that they received unpairings instead of six pairings for reconditioning. Note that both unpaired groups showed low freezing, indicating a low CS-US association, and that unpairings in the extinction context (unpaired group 2) did not induce significant long-term reinstatement of associative fear memory. A set of trained rats was tested for conditioned freezing on day 7, while another separate set was sacrificed on day 7 to prepare brain slices.

**Figure 2:**
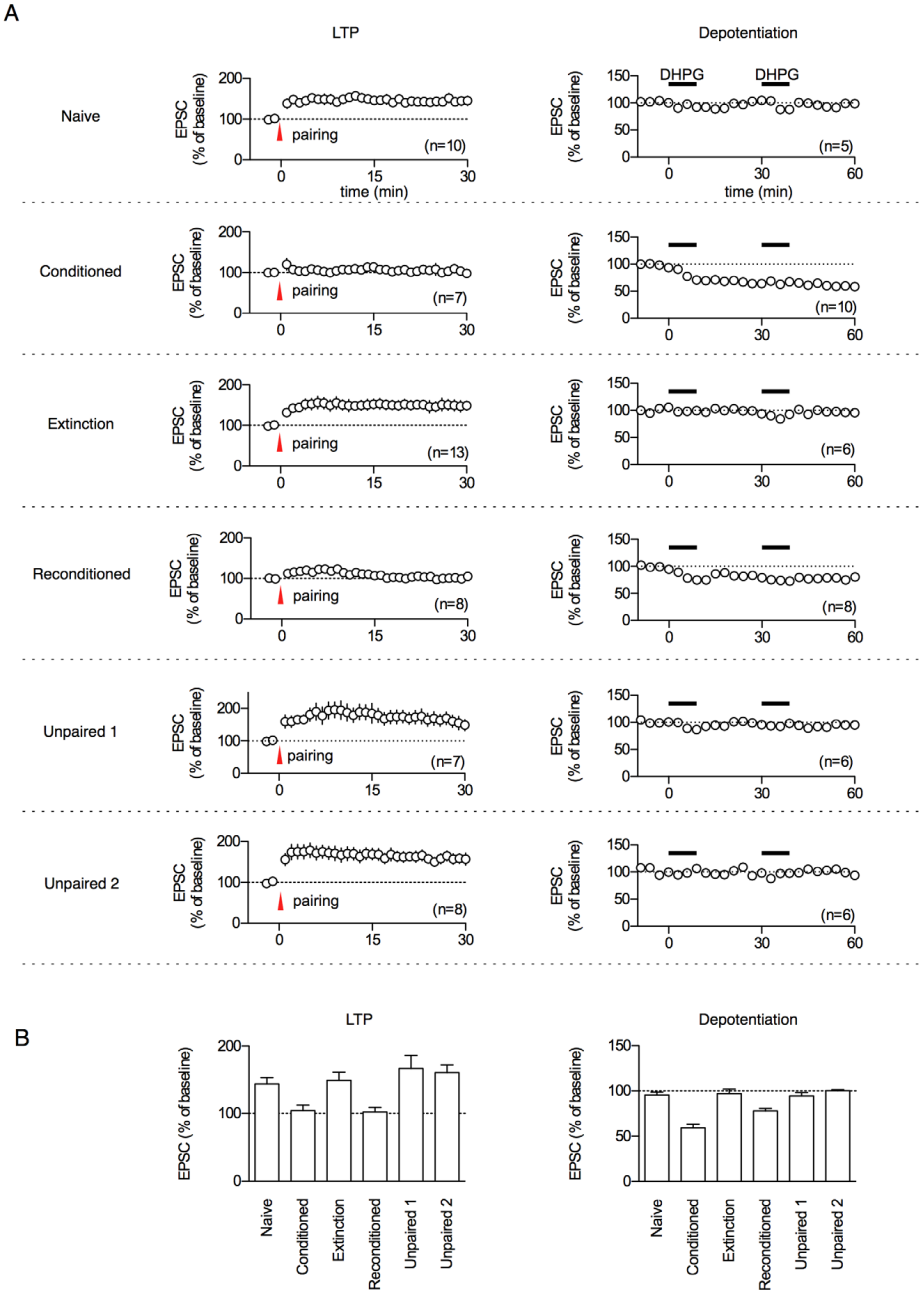
Reversible modification of T-LA synaptic efficacy within a fixed modification range. **A**. **Left**, Ceiling of the behaviorally modifiable range assessed with pairing-induced LTP. Robust LTP was observed in the groups for which T-LA synaptic weights were predicted to be at the floor of the range (naïve, extinction, unpaired group 1 & 2), whereas LTP was occluded in the groups for which T-LA synaptic weights were expected to be at the ceiling (conditioned, reconditioned). **Right**, Floor of the range estimated with depotentiation. Depotentiation was observed in the groups for which T-LA synaptic weights were predicted to be at the ceiling (conditioned, reconditioned), whereas depotentiation was absent in the groups for which T-LA synaptic weights were expected to be at the floor (naïve, extinction, unpaired group 1 and 2). **B.** Summary of the results shown in Fig. 2A. To avoid possible bias, the experiments in Fig. 2A were performed with the experimenter blind to the behavioral group.

In some instances, electrical stimulation-induced synaptic plasticity in brain slices such as LTP, long-term depression (LTD) and depotentiation are known to share mechanisms with learning-induced synaptic plasticity *in vivo* as evidenced by the occlusion of electrical stimulation-induced plasticity in brain slices with learning *in vivo* [19], [22], [23], [24]. This occlusion effect is possible since induction of both electrical stimulation- and learning-induced plasticity are saturable processes. Together, these previous results suggest the presence of a fixed modification range of at least some forms of learning-induced plasticity with upper and lower limits ([22] but see [25]). In fact, auditory fear conditioning is known to occlude LTP at cortical input synapses onto the LA, another important circuit for fear memory [23], [24], but whether conditioning occludes LTP at T-LA synapses has not been shown. We thereby tested whether fear conditioning would occlude LTP at the majority of T-LA synapses. If so, we further determined whether synaptic efficacy at the conditioning-modified T-LA synapses reversibly shifts between its ceiling and floor within a fixed modification range, predicting less LTP and enhanced depotentiation near the ceiling and enhanced LTP and less depotentiation near the floor. Synapses in the naïve and extinction groups were expected to be near the floor of the range, whereas synapses in the conditioned and reconditioned groups were expected to be near the ceiling. In addition, synapses in the unpaired controls for both the conditioning and reconditioning groups were expected to be near the floor of the range. The LTP induction protocol was delivered 5 min after the start of each whole-cell recording; the protocol failed induce LTP when applied >5 min after the start of the whole cell recording possibly due to a washout effect (data not shown; see [26]). During the first 3 min after the start of the whole-cell recording, the amplitude of the baseline responses was set to 100∼200 pA (average 157.42±11.88 pA). Data points collected from 3 to 5 min after the start of the recordings were used as a baseline, and recordings that showed a baseline drift of >10% were discarded. To test stability of the recordings, synaptic responses were collected for >30 min without any treatments. Under this condition the synaptic responses were found to be stable relative to the baseline (data not shown). As predicted, no significant LTP was found in either the conditioned or reconditioned group (groups at the ceiling: conditioned, 104.3±8.1%; reconditioned, 102.2±7.2%; p>0.05 for both groups, paired t-test), and enhanced LTP was observed in the naïve controls, extinction groups and two unpaired controls (groups at the floor: naïve, 143.7±9.6%; extinction, 149.3±12.0%; unpaired 1, 166.5±19.4%; unpaired 2, 160.3±11.6%; Fig. 2A and 2B). ANOVA indicated a main effect of group (F_5.47_ = 4.69, p = 0.0015), with post-hoc tests confirming that the magnitude of LTP was significantly higher in these four groups at the floor than in the conditioned and reconditioned groups (p<0.05 for all designated pairs, Newman-Keuls posttest), and that the magnitude of LTP did not differ significantly among the four groups at the floor (p>0.05 for all designated pairs, Newman-Keuls posttest). The presence of full LTP in the two unpaired controls indicates that the effects of conditioning and reconditioning on T-LA synapses are specific to associative learning-induced changes. The extent of LTP showed high negative correlation with fear memory strength (Pearson r = -0.9511, p = 0.0035, freezing vs. LTP level, for 6 pairs: naïve, conditioned, extinction, reconditioned, unpaired 1, unpaired 2).

The same set of experiments was performed with depotentiation. Depotentiation induced by the group I mGluR agonist DHPG (RS-3,5-dihydroxyphenylglycine) is known to detect conditioning-induced synaptic potentiation after memory consolidation [19], [27]. Results with LTP predict enhanced depotentiation in the conditioned and reconditioned groups, and less depotentiation in naïve, unpaired and extinction groups. DHPG-induced depotentiation (100 µM DHPG for 10 min) was induced twice, to verify floor of modification. Robust depotentiation was found in the conditioned and reconditioned groups (groups at the ceiling: conditioned, 59.1±4.3%; reconditioned, 78.0±2.8%), and no significant depotentiation was observed in the naïve, extinction and unpaired groups (groups at the floor: naïve, 95.4±3.5%; extinction, 97.1±4.9%; unpaired 1, 94.4±3.6%; unpaired 2, 100.4±1.2; p>0.05, paired t-test; Fig. 2A and 2B). ANOVA indicated a main effect of group (F_5,35_ = 20.78, p<0.0001), with post-hoc tests confirming that the magnitude of depotentiation was significantly higher in the conditioned and reconditioned groups than in the other four groups (p<0.01 for all designated pairs, Newman-Keuls posttest), and that the magnitude of depotentiation was significantly lower in the reconditioned groups than in the conditioned groups (p<0.01, Newman-Keuls posttest), which may reflect a small proportion of non-reversible plasticity with repeated use. Again, the extent of depotentiation held strong correlation with fear memory strength (Pearson r = 0.9290, p = 0.0074, freezing vs. depotentiation level, for 6 pairs: naïve, conditioned, extinction, reconditioned, unpaired 1, unpaired 2). Collectively, our findings suggest that synaptic efficacy in the majority of T-LA synaptic circuits can be reversibly modified between the maximum and minimum of a fixed modification range.

**Figure 3:**
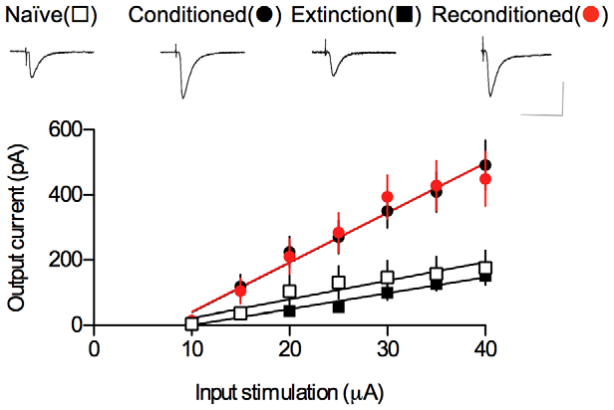
T-LA synaptic strength is reversibly modulated by conditioning, extinction, and re-conditioning. Input-output curves for EPSCs in naïve (n = 6), conditioned (n = 11), extinction (n = 8) and reconditioned (n = 9) groups. The series resistance was not significantly different between the four groups (naïve, 11.94±0.05 MΩ; conditioned, 12.13±0.10 MΩ; extinction, 12.07±0.10 MΩ; reconditioned, 12.05±0.10 MΩ; F_(3,34)_ = 0.70, p = 0.56; p>0.05 for all pairs, Newman-Keuls posttest). Decay time constants with input stimulation of 25 µA were not significantly different between the four groups (naïve, 5.12±0.48 ms; conditioned, 5.37±0.30 ms; extinction, 5.50±0.66 ms; reconditioned, 5.03±0.43 ms; F_(3,34)_ = 0.21, p = 0.89; p>0.05 for all pairs, Newman-Keuls posttest). Representative current traces are an average of four consecutive responses with input stimulations of 35 µA. Scale bars, 50 ms and 150 pA.

We next compared the input–output relationships for the EPSC amplitude as a function of afferent fiber stimulus intensity among four groups (naïve, conditioned, extinction and reconditioned groups). EPSCs were potentiated and reduced to the baseline in the conditioned and extinction groups relative to naïve controls, respectively, and they were fully re-potentiated in reconditioned groups relative to naïve controls and conditioned groups (naïve, 5.73±1.84 pA/µA; conditioned, 15.30±2.00 pA/µA; extinction, 4.87±0.64 pA/µA; reconditioned, 15.26±2.56 pA/µA). ANOVA indicated a main effect of group (F_3,30_ = 8.04, p = 0.0004), with a post-hoc test confirming that the slope of the input–output curve was significantly steeper in the conditioned and reconditioned groups than in the extinction groups and naïve controls (p<0.05 for all pairs, Newman-Keuls posttest), and that the slope of the input–output curve in the reconditioned and extinction groups did not differ significantly from that in the conditioned and naïve groups, respectively (p>0.05 for all designated pairs, Newman-Keuls posttest; Fig. 3). The fear memory strength as measured by freezing showed immense correlation with synaptic strength (Pearson r = 0.9990, p = 0.0010, freezing vs. input-output curve slope, for 4 pairs: naïve, conditioned, extinction, reconditioned).

## Discussion

In the present study, we have provided evidence that even after memory consolidation, initial memory-encoding T-LA synaptic circuits can be reversibly modulated by extinction and reconditioning. Initial conditioning appears to recruit the majority of T-LA synaptic circuits with learning-induced synaptic potentiation as evidenced by no significant LTP and enhanced depotentiation in brain slices after the conditioning. Extinction fully reverses the conditioning-induced changes; that is, no significant depotentiation and enhanced LTP, whereas re-conditioning reinstates the conditioning-induced changes (i.e., no significant LTP and enhanced depotentiation). Together these findings suggest the upper and lower limits of a fixed modification range for fear learning-induced reversible plasticity. Consistently, T-LA synaptic efficacy is enhanced with conditioning, reversed with extinction and reinstated with re-conditioning.

Our present findings support the presence of a fixed modification range of fear learning-induced plasticity even after memory consolidation. However, it should be noted that this apparently fixed range of the majority of T-LA synapses was estimated by two examples of synaptic plasticity. Thus, it is possible that other plastic or metaplastic mechanisms are still viable even in the upper and lower limits of a fixed modification range tested herein [25], [28]. It would be more appropriate to conclude that at least the plastic mechanism recruited by plasticity induction protocols used here can be reversibly modified after memory consolidation, and that other additional forms of plasticity and metaplasticity may operate to deal with other important facets of auditory fear memory.

Our results are in close agreement with several *in vivo* recording studies [29], [30], [31] that show an increase in amygdala response to conditioned stimuli after fear conditioning and decrease after fear extinction. We attribute these changes of response to the direct modulation of T-LA excitatory synaptic strength, and further extend the observation to subsequent re-learning. While the net synaptic strength observed here and in previous studies regarding the C-LA pathway [20] shows largely reversible modification, minute changes in individual synaptic weights may persist after fear extinction. Together with reports that fear memory can relapse even after extensive extinction [32], our data predicts that fear memory persists in a form other than the excitatory synaptic potentiation at T-LA synapses, perhaps in other brain regions [33], [34], [35] or as metaplastic mechanisms [36]. Although T-LA synaptic strength shows tight correlation with fear memory and explains most of the variance in freezing behavior among behavioral groups, other mechanisms (including new learning) and brain regions are well known to contribute to fear memory modulation. For instance, fear extinction is known to involve inhibitory mechanisms (engaging on both local and ITC inhibitory networks in the amygdala; [37], [38], [39]) and the medial prefrontal cortex (mPFC [40], [41], [42], [43], [44]). Interestingly, a recent study has shown that mPFC stimulation during extinction may enhance T-LA depotentiation [45]. Together, these findings suggest that multiple traces representing different facets of fear memory interact to modulate fear memory strength.

While a number of immuno-histological studies of fear memory encoding in the LA have suggested that only a small subpopulation of cells actually participate [46], [47], our present electrophysiological results, along with numerous others [13], [19], [20], [23], [48], [49], [50], have shown a clear increase of synaptic efficacy in considerably larger proportions of recorded cells. One reason why such discrepancy might emerge is that amygdala projection neurons are strongly inhibited by GABA-releasing local interneurons [51], [52], [53], displaying lower basal firing rates compared to other brain regions [54], [55]. This inhibition may obscure the expression of activity-dependent genes (e.g. pCREB, c-fos, Arc) used as a marker for memory traces in immuno-histological studies, resulting in a much smaller estimate of memory-encoding neurons. Indeed, several studies have shown that synaptic and neuronal plasticity are not restricted to neurons marked by these methods [56], [57]. It is also possible that the measurement of pCREB or c-fos immunoreactivity is thresholded so that subthreshold changes are lost; hence, these measurements could be dependent on the sensitivity of the immunological staining method. In contrast, electrophysiological measurements are made on a continuous scale, possibly enabling the detection of more minute synaptic modifications. In any case, the population-wide modulation of synaptic strength and occlusion of plasticity that we observe here suggests that fear conditioning to a particular auditory stimulus may recruit observable synaptic changes in a majority of LA neurons. This can be ascribed to the less frequency-tuned regions of the auditory thalamus that provide input to LA (the non-lemniscal auditory nucleus, including MGm and PIN [58], [59]) and/or receptive field growth to conditioned stimuli observed in these regions [60], [61], [62].

Our results suggest that learning-induced synaptic plasticity in the LA is functionally reversible and saturable, laying various implications both biologically and theoretically. The candidate mechanisms of LTP in the amygdala, including postsynaptic AMPAR trafficking [13], [63], altered presynaptic function [23], [48], spine enlargement [64], [65], [66], and PKMzeta activity [67], [68], [69], among others, may be subject to reversal upon depotentiation. This constraint of reversibility suggests that molecular mechanisms regulating LTP-related modifications are bi-directional, opening a new avenue to studies related to the down-regulation of synaptic function [70], [71], [72], [73], [74], [75]. Indeed, our results provide a robust model to test the *in vivo* functional relevance of various molecules required for depotentiation (and LTD), such as mGluRs [71], [76], Arc/Arg3.1 [77], [78], AP2 [79], PICK1 [80], PKC [81], Rap [82], [83], p38 [84], [85], Tyrosine phosphatases [86], GIRK [87], PP1 [88], and nNOS [89]. Genetic mouse models lacking such key molecules may exhibit abnormal fear extinction phenotypes if not impaired amygdala depotentiation.

Interestingly, the LTP and DHPG-induced LTD (depotentiation) measured in this study are known to oppositely involve the insertion and internalization of post-synaptic GluR2-containing AMPA receptors, respectively [71], [90], [91], [92]. Therefore the floor and the ceiling of synaptic modification (i.e. the consolidated memory-encoding portion of AMPA responses) defined in this study likely reflect a post-synaptic mechanism involving GluR2. These results support the ‘slot’ hypothesis [90], where the maximum synaptic expression of GluR2-containing AMPARs is constrained by postsynaptic slot molecules. Identification and verification of such rate-limiting molecule(s) may be expedited by monitoring expression of post-synaptic proteins in the LA during the behavioural protocol we have devised here. Thus our results provide a prominent model to study the learning-induced LTP/depotentiation mechanisms recruited *in vivo*.

The dynamic reuse of synapses shown in this study insinuates flexible network mechanisms of memory storage in the LA even after memory consolidation. This finding is in good agreement with studies involving artificial neural network models, where on-going alterations in connection weights are required if a network is to retain previously stored material while learning new information [93]. Experimentally, retention of consolidated memory has been shown to require recurrent activation of NMDA receptors [94]. Our results thus provide a hint to the “stability/plasticity dilemma” [95], suggesting that the regulated balance of synaptic stability and synaptic plasticity among different brain regions may support optimal memory performance of neuronal circuits.

## Materials and Methods

### Behavioral procedures

All procedures were approved by the Institute of Laboratory Animal Resources of Seoul National University (SNU-100503-5). Male Sprague-Dawley rats (4–5 weeks old) were maintained with free access to food and water under an inverted 12/12 hr light/dark cycle (lights off at 09:00 hrs). Behavioral training was done in the dark portion of the cycle. For fear conditioning, rats were placed in a conditioning chamber (San Diego Instruments, CA) and were left undisturbed for 2 min. Then, a neutral tone (30 s, 2.8 kHz, 85 dB SPL) co-terminating with an electrical foot shock (1.0 mA, 1 s) was presented three times at an average interval of 100 s. Note that the intensity of the auditory stimuli surpasses the known threshold distribution of most auditory thalamus neurons in the MGm/PIN, which relay auditory information to the LA [60]. For maximal conditioning, the three tone-shock pairings were repeated on the next day. Rats were returned to their home cage 60 s after the last shock had been applied. A Plexiglas chamber distinct from the conditioning chamber was used for both extinction training and tone tests. During extinction training, rats were presented with 20 tone presentations on the first day and 15 tone presentations on the following days at an average interval of 100 s without foot shocks, beginning 4 min after being placed in the chamber. The reconditioning procedure followed the protocol for maximal conditioning. Conditioned freezing was defined as immobility except for respiratory movements and was quantified by trained observers that were blind to the experimental groups. Total freezing time during a test period was normalized to the duration of either tone presentation (30 s) or context exposure. The final tone test was a single CS.

### Slice preparation

Brain slices were prepared using techniques described previously [19], [96]. In brief, Sprague-Dawley rats (3–5 weeks old) were anesthetized with halothane and decapitated. The isolated whole brains were placed in an ice-cold modified artificial cerebrospinal fluid (aCSF) solution containing (in mM) 175 sucrose, 20 NaCl, 3.5 KCl, 1.25 NaH_2_PO_4_, 26 NaHCO_3_, 1.3 MgCl_2_, 11 D-(+)-glucose, and gassed with 95% O_2_/5% CO_2_. Coronal slices (300 µm) including the LA were cut using a vibroslicer (HA752, Campden Instruments, Loughborough, UK) and were incubated in normal aCSF containing (in mM) 120 NaCl, 3.5 KCl, 1.25 NaH_2_PO_4_, 26 NaHCO_3_, 1.3 MgCl_2_, 2 CaCl_2_, 11 D-(+)-glucose and continuously bubbled at room temperature with 95% O_2_/5% CO_2_. Just before transferring a slice to the recording chamber, the cortex overlying the LA was cut away with a scalpel so that, in the presence of picrotoxin, cortical epileptic burst discharges would not invade the LA. DHPG was obtained from Tocris Bioscience; all other chemicals were obtained from Sigma-Aldrich (St Louis, MO, USA). DHPG was dissolved in a stock dH_2_O solution (100 mM) freshly every week and was diluted at 1∶1000 for treatment.

### Recording conditions

Whole-cell recordings were performed from visually identified pyramidal neurons in the dorsolateral division of the LA. The cells were classified as principal neurons based on the pyramidal shape of their somata. While voltage-clamped, a minor proportion (<5%) of recorded neurons exhibited spontaneous excitatory postsynaptic currents (EPSCs) with faster decay times and larger amplitude (>100 pA), characteristics typical of interneurons in the LA [97], and were excluded from analysis (see also Supplementary Fig. 7 in [19]). We included picrotoxin (100 µM) in our recording solution to isolate excitatory synaptic transmission and to block feed-forward GABAergic inputs to principal neurons in the LA.

### Afferent stimulation

We chose brain slices containing a well-isolated, sharply defined trunk (containing thalamic afferents) innervating the dorsolateral division of the LA, where somatosensory and auditory inputs are known to converge [98]. The sizes of the LA and central amygdala were relatively constant in these slices, and the closest trunk to the central nucleus of the amygdala was used when multiple trunks were observed. Thalamic afferents were stimulated using a concentric bipolar electrode (MCE-100, Rhodes Medical Instruments, CA) placed on the midpoint of the trunk between the internal capsule and medial boundary of the LA (see also Fig. 1 in [19]). Regions and cells of interest for all recordings were located beneath the midpoint of the trunk spanning the LA horizontally.

### Whole-cell patch-clamp recordings

Whole-cell recordings were made using a MultiClamp 700A (Molecular Devices, CA). Recordings were obtained using pipettes with resistances of 2.5–3.5 Mohm when filled with the following solution (in mM): 100 Cs-gluconate, 0.6 EGTA, 10 HEPES, 5 NaCl, 20 TEA, 4 Mg-ATP, 0.3 Na-GTP, and 3 QX314; with the pH adjusted to 7.2 with CsOH and osmolarity adjusted to around 297 mmol/kg with sucrose. Recordings were made under IR-DIC-enhanced visual guidance from neurons that were three to four cell layers below the surface of the 300-µm-thick slices at 32.5±0.5°C. Neurons were voltage-clamped at −70 mV, and solutions were delivered to slices via superfusion driven by gravity at a flow rate of 1.5 ml/min. The pipette series resistance was monitored throughout the experiments, and if it changed by >20%, the data were discarded. Whole-cell currents were filtered at 1 kHz, digitized at 20 kHz, and stored on a microcomputer (Clampex 8 software, Molecular Devices). Pairing-induced LTP was induced by 15 bursts of presynaptic stimuli, with each burst consisting of three stimuli delivered at 30-ms intervals (an interburst interval of 5 s), while a postsynaptic neuron was held at 0 mV throughout the duration of all bursts. Due to washout effect [26], LTP was induced 5 min after achieving whole-cell configuration in all cells, and the last 2 minutes before LTP induction was used as baseline. One or two neurons were recorded per animal (a single neuron per slice). All recordings were completed within 4 hrs after slice preparation, mainly due to cell viability of the 300-µm-thick slices. For better display, running averages of four or eight data points were applied in the time-lapse experiments.

### Statistical analysis

The results comparing single data points between behavior-trained groups were analyzed with an unpaired t-test (for comparison of two treatment groups) or one-way ANOVA with subsequent Newman-Keuls post hoc comparison (for more than two treatment groups). In several experiments, the paired t-test was used to determine whether synaptic responses after plasticity induction differed significantly from baseline responses. In the plasticity experiments, a temporal average of the data points during a period of interest was used for statistical comparison of EPSC (12 min) results. Linear correlation was measured with the Pearson product moment correlation coefficient, treating each behavior group as a single sample, as behavioral and electrophysiological observations were made in separate animals. A probability value of p<0.05 was considered indicative of statistical significance.
